# An unusual presentation of pancreatic pseudocyst mimicking cystic neoplasm of the pancreas: a case report

**DOI:** 10.1186/1757-1626-2-9138

**Published:** 2009-12-03

**Authors:** Aleksandra Gintowt, Stanislaw Hac, Sebastian Dobrowolski, Zbigniew Śledziński

**Affiliations:** 1 Department of General, Endocrine and Transplant Surgery, Medical University of Gdansk, 7 Dębinki Street, 80-952 Gdańsk, Poland

## Abstract

In spite of their rarity, cystic neoplasms of the pancreas are characterized by existing or potential malignancy that cannot be ignored during decisive process with regard to the choice of treatment. Diagnostic difficulties in the differentiation of pancreatic pseudocyst and cystic pancreatic neoplasm can lead to misdiagnosis and inappropriate treatment, since clinical symptoms, preoperative imaging tests and even endoscopic retrograde cholangiopancreatography are often not sufficient to establish the correct diagnosis. We present a case of pancreatic cyst with no typical features of pseudocyst in the medical interview, treated by Child's subtotal pancreatectomy by reason of the high risk of neoplasia suggested by radiological and endoscopic examinations.

## Introduction

Cystic tumors account for 10% of all pancreatic cysts, but only 1% of all pancreatic malignancies [1]. One of the most important differences between cystic neoplasm and pseudocyst is its radiological picture and a history of abdominal trauma, acute or chronic pancreatitis [2]. Despite the advances, concerning preoperative diagnosis, in a lot of cases of pancreatic cystic lesions final diagnosis could be established only after excision. Cystic tumors of the pancreas can be divided into relatively common, such as serous microcystic adenoma [3], intraductal mucinous tumor [3], mucinous cystic tumor [3] and solid pseudopapillary tumor [4] and uncommon, such as cystic endocrine tumors [5], cystic metastasis [6], cystic teratomas [7] and lymphangiomas [8]. Many types of cystic pancreatic neoplasms require management by pancreatic resections, therefore early recognition and surgical treatment play an important role. The main problem considered in our case is differentiation of pancreatic pseudocysts and cystic neoplasms. In such cases only histological examination after surgical resection can establish the diagnosis.

## Case presentation

We present a case of pancreatic cyst without a history of trauma or panceratitis. A Polish Caucasian 38-year-old woman was admitted to the University Hospital in 2004 because of erythrocyturia presented in routine urine analysis. Patient occasionally felt left-sided abdominal pain occurring after abstaining from eating with an accompanying weakness retiring after eating sweets. For 2-3 months she occasionally observed nausea after meals.

**Figure 1:**
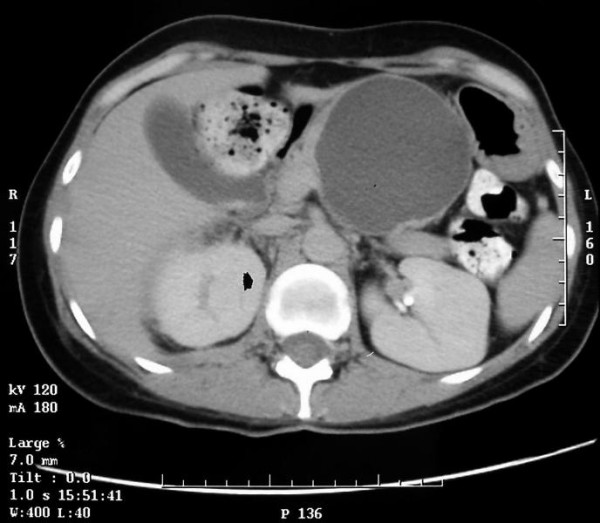
**CT scan showing large cystic lesion of the pancreas**. Thin walled lesion without segmentation with homogenous fluid content.

**Figure 2:**
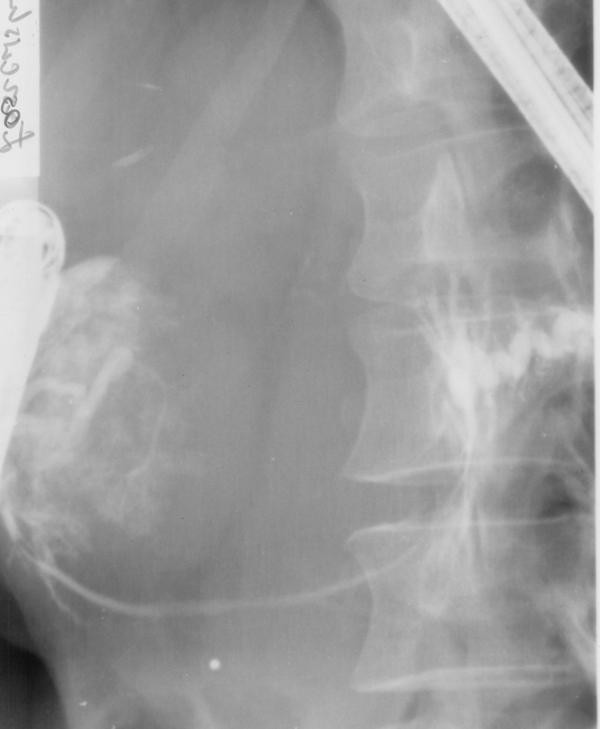
**ERCP showing displaced pancreatic duct without cyst communication**.

Patient underwent an abdominal ultrasound and computed tomography (CT) that revealed normal-sized liver and spleen, both without any focal changes and a gall bladder devoid of shading stones with visible polyps. Between the left lobe of the liver, stomach and the body of the pancreas there was thin walled pancreatic cyst with a diameter of 8 cm and a density of 9 HU (Figure 1). The pancreas was narrow with conspicuous Wirsung's duct within the body and the tail. The size of the head was normal. There were no enlarged lymph nodes. In organs of the small pelvis one couldn't see any changes in spite of a small amount of liquid. Thereafter, patient was transferred to Department of Gastroenterology. Endoscopic retrograde cholangiopancreatography (ERCP) examination revealed no communication between the cyst and the pancreatic duct (Figure 2). It also showed impression on the antrum of the stomach and the swollen papilla of Vater. In the head of the pancreas the main pancreatic duct was unchanged, from the neck to the body of the pancreas it was pressed and displaced by a tuberous structure (the wall of the duct was smooth with no secondary branches), in the tail the duct was extended and intricate. Pancreatic sphincterotomy was performed and only the tray was introduced to the tail. As the conclusion, endoscopist suggested pancreatic cystadenoma. Three days after ERCP patient presented fever, right-sided abdominal pain and redness in the region of the right kidney with positive Goldflam's sign. Next abdominal ultrasonography and CT revealed right pararenal inflammation with suggestion of abscess formation. The reason of inflammation remained unclear, but the probable relation to ERCP examination is conceivable. In March 2004 patient underwent successful incision and drainage of the abscess located around the right kidney. Urine culture revealed negative results. Patient was treated with ciprofloxacin and methronidazole.

**Figure 3:**
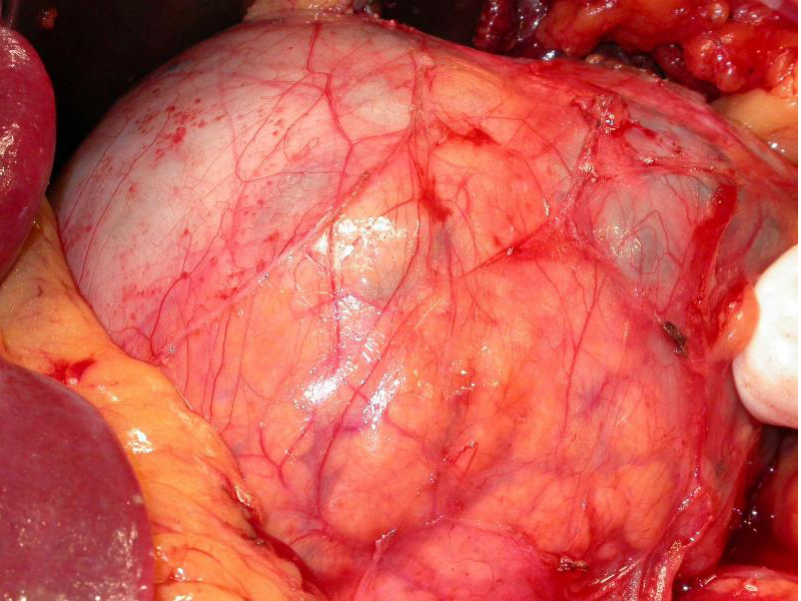
**Pancreatic cyst located in the body of pancreas**.

**Figure 4:**
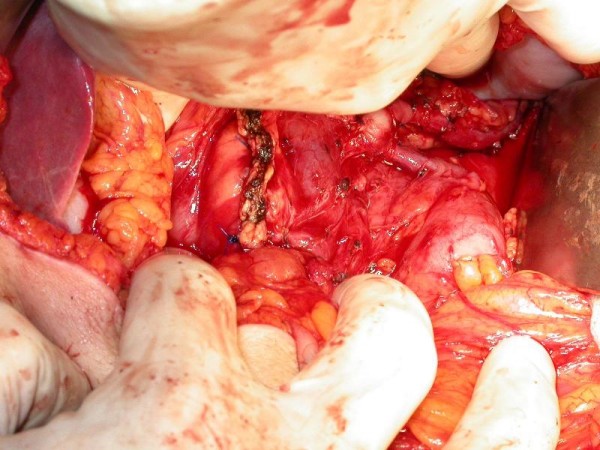
**State after surgical resection of the cyst**.

After control CT that confirmed right pararenal abscess, complete healing Child's subtotal pancreatectomy and cholecystectomy were performed by open way. In the neck of the pancreas polycyclic, encapsulated, not connate to the environment formation was found (Figure 3). On account of the solid concrescence of the cyst and the portal vein, during the preparation over the vessel, lesion of its front wall (width: 0.5 cm) took place. The defect was sutured with leaving the fragment of the cyst wall on the portal vein (Figure 4). There were no macroscopic indications to qualify cyst as malignant. Afterwards the remaining part of the cyst was prepared.

The post-operative course was uneventful. Patient was discharged home within 7 days in a good general condition. There were no symptoms of glucose intolerance after normal diet administration.

The microscopic examination revealed considerable fibrosis and dispersed lymphocyte swelling in the pancreatic cyst. Tissue samples of the pancreas presented chronic non-specific pancreatitis with fibrosis. In the gallbladder chronic non specific high grade cholecystitis was recognized.

Patient was visited at home in 2007. She felt well, with no severe complaints and with correct periodic findings and normal glucose metabolism.

## Discussion

In most of cases of pancreatic fluid collections radical surgery is not necessary. Pseudocysts require drainage via the papilla, if they connect with the pancreatic duct, internal drainage (cystogastrostomy or Roux-en-Y) or cystoduodenostomy, CT or echo guided drainage [9,10]. If their size reaches 6 cm or less, they do not need any treatment. In our case the diameter of the cyst was 8 cm and there were no episodes of acute pancreatitis in the medical interview. Presented case was classified by experienced endoscopist and radiologist as cystadenoma or cystic pancreatic tumor. No symptoms in the physical examination were manifested. Misdiagnosing the neoplasm and using drainage instead radical surgery can lead to complications, therefore any serious risk could not be ignored. Long term survival for completely removed mucinous cystadenocarcinomas is over 60% [11], but very serious in invasive ones. In MCNs, recognition of an underlying malignancy is often not possible without microscopic examination of the whole specimen.

The clinical manifestation of cysts and cystic tumors can be identical or they often can be asymptomatic. Symptoms as abdominal discomfort or abdominal pain, nausea, vomiting, diarrhea, fever and leukocytosis, icterus, recurrence pancreatitis, bleeding from the digestive tract, abdominal mass are typical in both pseudocyst and neoplasm cases. In the history of presented patient there were no alcohol abuse nor biliary lithiasis. The aetiology of chronic pancreatitis in this case remain unknown, however similar events are described and idiopatic pancreatitis account for up to 25% cases of chronic pancreatitis [12]. The mass was thin walled and relatively soft. Patient was surprisingly asymptomatic, as usually in case of cystic pancreatic lesions in corpus and tail without communication with pancreatic duct. We found a case of an asymptomatic huge unilocular pseudocyst measuring 7 cm in a Japanese man [13].

CT is found to be a very helpful tool in detection of pancreatic tumors, with sensitivity over 90% (when direct and undirect signs are used for diagnosis), but is not an ideal tool for differentiation exocrine tumors of the pancreas [14]. Computed tomography is useful for detection a lesion, distinguishing the microcystic subgroup of serous cystadenoma and showing rim calcifications, but is not reliable for distinguishing neoplasm from pseudocyst, serous from mucinous tumors or benign from malignant [1]. Such distinctive finds as lack of solid components of the cystic wall, multiply loculations, peripheral calcifications, infiltration in surrounding tissue and polycystic structure of the lesion recognized as typical features of neoplasm are not always seen in some evident neoplasm cases. In our case (finally diagnosed chronic pancreatitis) there wasn't any clinical evidence of chronic pancreatitis calcifications as well in the image examinations, which may be found in only 30% of patients with chronic pancreatitis.

Preoperative tumors markers might be helpful in decision making and postoperative management of pancreatic neoplasms. Tumor markers were not controlled in described case because of negative opinion of senior gastroenterological consultant.

Tumor markers are used mainly in patients already diagnosed with cancer for monitoring their response to treatment. In our case indications for the operation was strong enough without controlling tumor markers, especially in the face of fact that in early stages of carcinoma levels of Ca 19-9 (the most promising marker) may not be elevated and in some diseases of liver or pancreas it also can be temporarily raised. Unfortunately, there is no ideal tumor marker for pancreatic carcinoma.

ERCP has been used for the diagnosis and treatment of pancreatic diseases for years. Some cystic tumors and 60% of pseudocysts are connected to the Wirsung's duct and this communication suggests a diagnosis of rather pseudocyst than cystic neoplasm. ERCP can show a communication of the lesion with the main pancreatic duct, but it isn't a perfect method for confirming or expelling the recognition of neoplasm. ERCP can help with establishing a tissue diagnosis of pancreatic neoplasms by: brush cytology, intraductal biopsy and fine needle aspiration, but because of low sensitivity of duct brushings and the potential morbidity connected with ERCP, this method is often replaced by endoscopic ultrasound (EUS is also powerful to show the precise internal structures such as mural nodules [15]). ERCP is useful to examine the cystic lesion, but does not visualize it when the lesion is filled with excessive mucin [15]. Intraductal papillary mucinous tumors can be recognized and differentiated from a pseudocysts or pancreatic cystic neoplasms using ERCP (opened ampulla's of Vater with mucous in IPMTs). Needle aspiration or biopsy results would be of potential diagnostic value, but our case there was no technical possibility of fluid aspiration during ERCP in opinion of gastroenterologist. On the other hand the result of a needle aspiration or a biopsy might be not always sufficient for the diagnosis of pancreatic cystic neoplasms. Concentration of amylase in liquid taken from the cyst, increased in pseudocysts, is insignificant in neoplasms. Although high amylase concentration suggests benign character of lesion, cyst fluid analysis may be misleading in an individual patient [16]. We also can't definitively expel neoplasm transformation of pancreatic pseudocyst. Cyst fluid analysis for tumor markers (carcinoembryonic antigen: CEA, CA 125), relative viscosity and cytology can credibly distinguish malignant cystic tumors and potentially premalignant mucinous cystic neoplasms from pseudocysts and serous cystadenomas, amylase content with isoenzyme analysis is useful to identify pseudocysts [17].

MRI (T2-weighted image) and magnetic resonance cholangiopancreatography are able to display the whole cystic lesion [15] with the similar diagnostic power as CT. Additional visualizing examinations (MRI scan, MRI angiography or colour Doppler investigation) might be of value in pancreatic pathology. Angiography nor MRI were not performed preoperatively because experienced radiologist estimate the operability on CT pictures. On the other hand decision of inoperability in radiological examination of pancreatic lesions because of vein involvement are overestimated in at least 25% of cases. Decision of inoperability of not disseminated pancreatic lesions are always made on operating room as routine.

In the presented case, strong suggestion of cystic tumor made by radiologist and endoscopist was taken into consideration in surgical treatment decision making. The pancreatic cyst was qualified preoperatively as pre or neoplasmatic one. On exploration there were no criteria of malignancy or suspicion of premalignant cystic tumor. Operating surgeon decide to preserve portal vein without resection leaving part of cystic wall. The idea of that was the resection of this part in case of malignancy after microscopic postoperative examination. The decision would be made on intraoperative examination but after consultation with pathologist there was no suspected place inside the cyst and the team decide to carefully analyze the cyst in routine manner. The microscopy confirmed benign character of the lesion thus patient underwent normal follow-up without radicalisation of surgery necessity.

## Conclusion

In our case, the risk of malignancy of the lesion was too high to observe the patient. The medical interview was atypical for pancreatic pseudocyst and diagnostic difficulties in differentiation between pseudocyst and cystic tumor exerted influence on the choice of operative intervention as the best treatment strategy. In consideration of imperfection of nowadays diagnostic tools we adjudged radical operative intervention as the reasonable and well documented method of treatment. In retrospective analysis presented case might be the chronic subclinical pancreatitis with post ERCP exacerbation and infection manifested as pararenal retroperitoneal abscess. Taking all together the better choice in doubtful cases of pancreatic cystic lesions seems to standardize diagnostic procedure.

## Consent

Written informed consent was obtained from the patient for publication of this case report and any accompanying images. A copy of the written consent is available for review by the Editor-in-Chief of this journal.

## Competing interests

The authors declare that they have no competing interests.

## Authors' contributions

AG analyzed and interpreted the patient and was the contributor in writing the manuscript. SH performed surgery, and was a major contributor in writing the manuscript. SD participates in data interpretation and contributes in manuscript writing. ZS supervise the whole case presentation and assist in discussion writing. All authors read and approved the final manuscript.
